# Utility of bevacizumab in advanced hepatocellular carcinoma: A veterans affairs experience

**DOI:** 10.1002/cam4.2015

**Published:** 2019-02-20

**Authors:** Max M. Wattenberg, Nevena Damjanov, David E. Kaplan

**Affiliations:** ^1^ Division of Hematology‐Oncology Perelman School of Medicine University of Pennsylvania Philadelphia PA, USA; ^2^ Hematology‐Oncology Section Corporal Michael J. Crescenz VA Medical Center Philadelphia PA, USA; ^3^ Division of Gastroenterology and Hepatology Perelman School of Medicine University of Pennsylvania Philadelphia PA, USA; ^4^ Gastroenterology Section Corporal Michael J. Crescenz VA Medical Center Philadelphia PA, USA

**Keywords:** bevacizumab, HCC, hepatocellular carcinoma, liver cancer, VEGF

## Abstract

Hepatocellular carcinoma (HCC) is a challenging to treat malignancy with few available systemic therapies. Angiogenesis has been implicated in the pathogenesis of HCC and prior studies have suggested a role for anti‐VEGF therapy. Prior to FDA approval of second‐line therapy for advanced HCC, from 2008 until 2017, we initiated bevacizumab monotherapy (5‐10 mg/kg every 2‐3 weeks) in 12 patients with intolerance of or progression during sorafenib therapy. Bevacizumab therapy was well tolerated with only 1/12 patients experiencing a grade 3‐4 treatment‐related adverse event (transient ischemic attack) and only 2/12 patients discontinued the therapy due to adverse events. Median overall survival was 20.2 months (IQR, 7.0‐43.5), with a median time to radiologic progression of 10.4 months (IQR, 2.8‐16.1) and a disease control rate of 54%. Taken together, our experience provides rationale for further prospective investigation of bevacizumab for the treatment of advanced HCC.

## 1 INTRODUCTION

There are an estimated 42 220 new cases of hepatocellular carcinoma (HCC) annually in the United States with an associated 5 year overall survival of less than 20%.^1^ Partly due to the lack of new effective therapies, HCC mortality rates have increased nationally over the last several decades.^2^ A paucity of systemic therapy options for advanced disease has been especially problematic. In 2008, Llovet et al, in a phase III randomized controlled trial (RCT), demonstrated the efficacy of sorafenib for the treatment of advanced HCC (HR 0.69; 95% CI, 0.55‐0.87; *P* < 0.001). Sorafenib is an oral multikinase inhibitor with activity against vascular endothelial growth factor (VEGF) signaling.^3^ Elucidation of the importance of angiogenesis in driving HCC biology provided further rationale for testing of anti‐VEGF therapies.^4^ Siegel et al conducted a phase II trial of bevacizumab, a recombinant humanized monoclonal antibody against VEGF, for the treatment of nonmetastatic HCC. The objective response rate (ORR) was 13% and median progression free survival (PFS) was 6.9 months (95% CI, 6.5‐9.1).^5^ Subsequently, several phase II trials studied bevacizumab in combination with erlotinib, capecitabine or multi‐agent chemotherapy and further demonstrated the potential of bevacizumab for the treatment of HCC.^6^, ^7^, ^8^ To date, bevacizumab for the management of HCC has not been studied in the phase III setting. Prior to 2017, sorafenib was the only US Food and Drug Administration (FDA) approved agent for the treatment of advanced HCC. Given the lack of available systemic therapies prior to 2017 and based upon phase II results and medication availability, we utilized bevacizumab off‐label as a second‐line agent for the treatment of patients with advanced HCC who progressed on or were intolerant of sorafenib. Herein we report our experience treating advanced HCC with bevacizumab.

## 2 METHODS

This retrospective study reviewed all patients with HCC treated with bevacizumab at the Corporal Michael J. Crescenz VA Medical Center (Philadelphia, PA) between August 2014 and July 2018. Patients were identified for inclusion by interrogation of the facility's Multidisciplinary Liver Cancer Tumor Board Database**.** Following patient identification, data were collected retrospectively from the VA computerized patient record system (CPRS). Inclusion criteria included: confirmed diagnosis of HCC by imaging (LiRADs) and/or biopsy, treatment with bevacizumab and age >18 years.^9^ Exclusion criteria included: treatment with bevacizumab for non‐HCC malignancy. Outcome measures were defined as follows: overall survival (OS) was defined as time from start of bevacizumab to death; time to radiological progression (TTRP) was defined as the time from start of bevacizumab to progression on imaging as defined by mRECIST^10^; disease control rate (DCR) was defined as the percentage of patients who had a best‐response rating of complete response (CR), partial response (PR) or stable disease (SD) at any time point while on treatment with bevacizumab. OS and TTRP were calculated using Kaplan‐Meier methodology in R.^11^, ^12^ This study was approved by the institutional review board (IRB) at the Corporal Michael J. Crescenz VA Medical Center (Philadelphia, PA) with a waiver of informed consent.

## 3 RESULTS

### 3.1 Patients

Between August 8th, 2014 and July 24th, 2018, there were 12 patients with advanced HCC treated with bevacizumab. The patient characteristics were largely representative of the veteran liver cancer population (Table 1). The median age of the patients was 62 years (range, 55‐71) and all patients were male. The majority (66%) of the patients were black and the remaining patients were white. Chronic hepatitis C (HCV) was the major risk factor for the development of HCC. Of 10 patients with HCV, five had concurrent alcoholic liver disease. Other etiologies of chronic liver disease included hemochromatosis and chronic liver disease of unknown etiology. All patients had an ECOG performance status of 0 or 1. Underlying liver disease was generally well compensated and most patients were Child‐Pugh class A (83%). Biochemical analysis was also consistent with compensated liver disease. The median albumin level was 3.2 g/dL (range, 2.2‐4.2) and the median total bilirubin was 0.9 mg/dL (range, 0.5‐2.3). Median INR was 1.0 and was less than 1.3 in all patients. The median AFP was 15.1 ng/mL (range, 1.4‐7780). Barcelona Clinic Liver Cancer (BCLC) stage was B or C in 92% of patients. TNM stage ranged from II to IVB. Of the 12 patients, four were stage II, five were stage IIIA or B, and three were stage IVB. All patients had received prior locoregional therapy and the majority of patients (92%) had received prior systemic therapy. Locoregional therapy consisted of transarterial chemoembolization in all patients and, less commonly, surgical resection, radiofrequency ablation, and radiotherapy. The most common prior systemic therapy was sorafenib in 10 of the 12 patients. One patient received ipafricept on clinical trial prior to bevacizumab therapy.

**Table 1:** Demographic and baseline characteristics of the patients

Characteristic	Bevacizumab (N = 12)
Age – yr	62 ± 4.9
Sex ‐ no. (%)	
Male	12 (100)
Female	0 (0)
Cause of disease ‐ no. (%)	
Hepatitis C only	5 (42)
Alcohol only	0
Hepatitis C and alcohol	5 (42)
Other	2 (16)
ECOG performance status ‐ no. (%)^a^	
0	10 (83)
1	2 (17)
BCLC stage ‐ no. (%)^b^	
A	1 (8)
B	6 (50)
C	5 (42)
Child‐Pugh class ‐ no. (%)^c^	
A	10 (83)
B	2 (17)
Biochemical analysis	
Albumin ‐ g/dL	
Median	3.2
Range	2.2‐4.2
Total bilirubin ‐ mg/dL	
Median	0.9
Range	0.5‐2.3
Alpha‐fetoprotein ‐ ng/mL	
Median	15.1
Range	1.4‐7780
Previous therapy ‐ no. (%)	
Surgical resection	2 (17)
Locoregional therapy	
Transarterial chemoembolization	12 (100)
Radiofrequency ablation	2 (17)
Radiotherapy	1 (8)
Systemic anticancer therapy	
Sorafenib	10 (83)
Other	1 (8)
None	1 (8)

BCLC, Barcelona Clinic Liver Cancer Stage; ECOG, Eastern Cooperative Oncology Group; SD, standard deviation.

a Easter Cooperative Oncology Group Performance Status is a measure of functional status and ranges from 0 (asymptomatic) to 5 (death).

b Barcelona Clinic Liver Cancer Stage is a system that takes into account performance status, tumor burden and liver function and ranks patients from stage 0 (very early stage) to D (terminal stage).

c Child‐Pugh score is used to assess prognosis in chronic liver disease and ranks patients from class A to class C (end stage liver disease).

### 3.2 Outcomes

**Figure 1:**
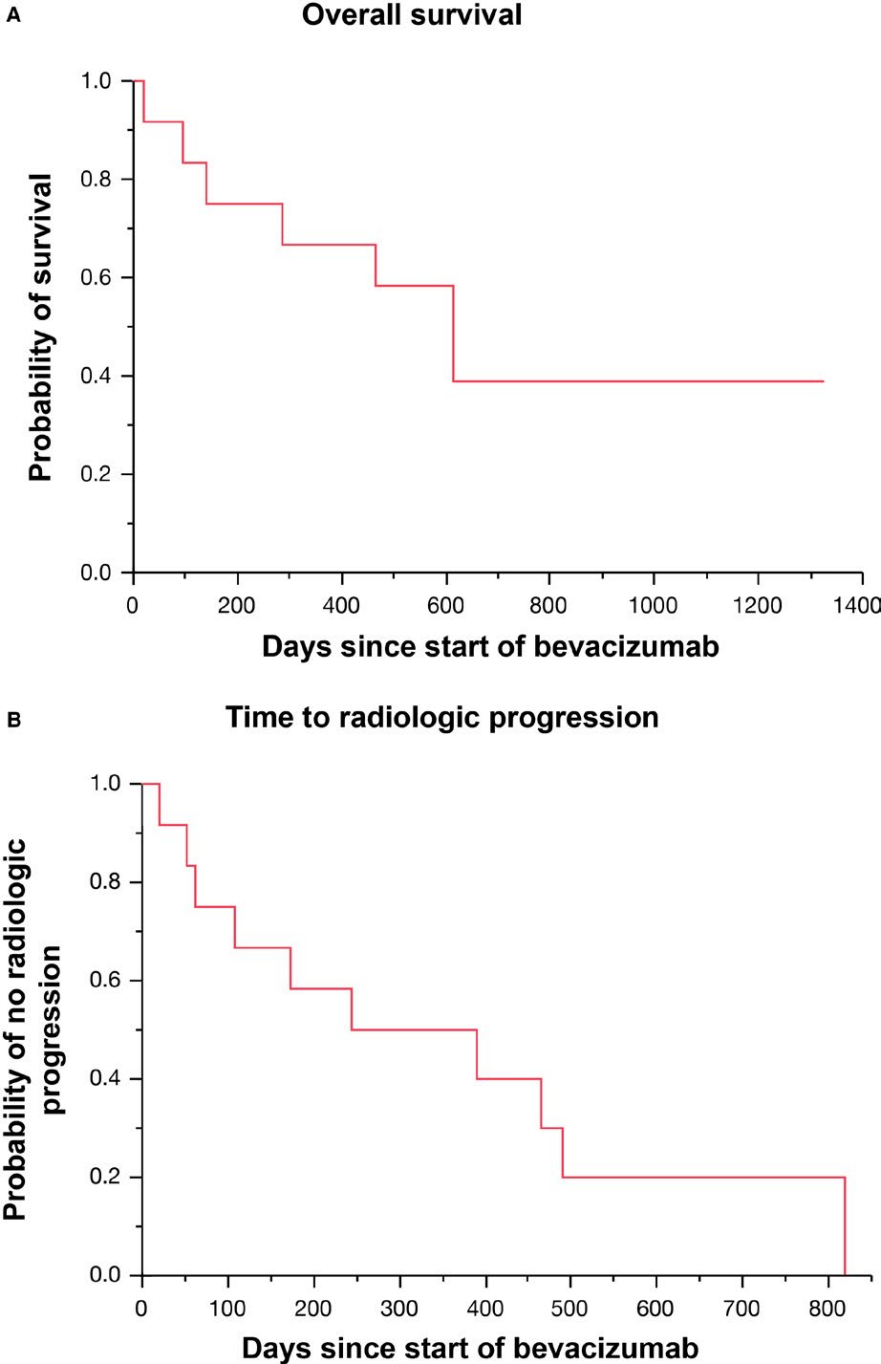
Kaplan‐Meier analysis of overall survival and time to radiologic progression. Of the 12 patients who were treated with bevacizumab, the median overall survival was 20.2 months (Panel A) and the median time to radiologic progression was 10.4 months (Panel B)

The median overall survival was 20.2 months (range, 0.7‐44.1). Five of the twelve patients remained alive at time of censoring with a median of 13.1 months of bevacizumab exposure. At the time of analysis 10 of the 12 patients had developed progression of disease by imaging or had died. Median time to radiologic progression was 10.4 months (range, 0.7‐27.3) (Figure 1). At time of analysis one patient had ongoing partial response. Of the 12 patients treated with bevacizumab, three achieved PR (27%) and three demonstrated SD (27%). No patients had a CR. The DCR was 54% (Table 2).

**Table 2:** Summary of efficacy measures

Outcome	Bevacizumab (N = 12)
Overall survival^a^	
Median (IQR)	20.2 (7.0‐43.5)
Time to radiologic progression (mo)^b^	
Median (IQR)	10.4 (2.8‐16.1)
Level of response (%)^c^	
Complete	0
Partial	27
Stable disease	27
Disease control rate (%)^d^	54
Duration of bevacizumab exposure	
Median (IQR)	8.5 (3.2‐14.3)

IQR, interquartile range.

a Overall survival was defined as time from start of bevacizumab to death.

b Time to radiological progression was defined as the time from start of bevacizumab to progression on imaging as defined by mRECIST.

c Level of response was measured according to mRECIST.

d The disease control rate was the percentage of patients who had a best‐response rating of complete or partial response or stable disease at any time point while on treatment with bevacizumab.

### 3.3 Treatment compliance

The median duration of treatment with bevacizumab was 8.5 months (range, 0.5‐42). The most common reason for discontinuation was progression of disease in 10 of the 12 patients. Two patients discontinued the treatment due to adverse events including transient ischemic attack in one patient and a combination of fatigue, anorexia, and nausea in a second patient. Bevacizumab was generally well tolerated in this cohort (Table 2). Bevacizumab dosing was at the treating physician's discretion. Four patients received 5 mg/kg every 2 weeks, four patients received 7.5 mg/kg every 3 weeks, and the remaining four patients received 10 mg/kg every 2 weeks.

## 4 DISCUSSION

In this single‐institution retrospective case series, we describe our experience treating advanced HCC with bevacizumab. In this study of 12 patients treated with bevacizumab (5‐10 mg/kg every 2‐3 weeks), the median OS was 20.2 months and the time to radiologic progression was 10.4 months. Although the small study size and heterogeneous population limit definitive conclusions, the clinical benefit in this retrospective analysis was greater than expected based on historical phase II data.^5^ The vast majority of patients were Child‐Pugh class A with performance status of 0‐1 at time of treatment, likely contributing to the excellent outcomes. However, all patients had advanced HCC (including eight patients with stage III or IV disease) and all had been heavily pretreated with a combination of local and systemic therapy prior to receiving bevacizumab. The majority of patients were HCV‐infected suggesting bevacizumab is safe in HCC associated with viral hepatitis. Taken together, these findings provide rationale for the continued study of bevacizumab in the treatment of patients with advanced HCC.

Prior to April 2017, sorafenib represented the only systemic agent FDA approved for the treatment of HCC. Subsequently, multiple advances have been made in the first and second‐line setting. In 2017, regorafenib, an oral agent with action against VEGF signaling, obtained FDA approval for the treatment of patients with advanced HCC after progression on sorafenib.^13^ In the first‐line setting, FDA approval was recently obtained for the oral multikinase inhibitor lenvatinib after a phase III trial demonstrated noninferiority as compared to sorafenib. Lenvatinib has action against VEGF receptors 1‐3, KIT, RET, FGFR, and PDGFR.^14^

Additional agents targeting VEGF signaling are in development. Ramucirumab is a human monoclonal antibody against VEGF receptor 2. The phase III trial REACH‐2 studied ramucirumab in patients with advanced HCC and AFP greater than 400 and showed significant improvement in OS as compared to placebo (HR 0.71; 95% CI, 0.53‐0.94; *P* = 0.0199).^15^ Cabozantinib is an oral multikinase inhibitor with activity against MET, AXL, and VEGF receptors 1‐3. MET and AXL have been implicated in resistance to sorafenib. The phase III CELESTIAL trial demonstrated improvement in OS with cabozantinib as compared to placebo in the treatment of patients with advanced HCC patients who progressed on sorafenib (HR 0.76; 95% CI, 0.63‐0.92; *P* = 0.005).^16^

Given the discussed findings, the efficacy of VEGF signaling inhibition in HCC treatment has been established. However, no comparative trials between available agents have been performed. Bevacizumab in the phase II setting and in our study showed similar or better OS as compared to ramucirumab, cabozantinib, and regorafenib. In addition, the cost‐effectiveness of bevacizumab has improved significantly with FDA approval of the first bevacizumab biosimilar.^17^ Further study is needed to determine the safety and efficacy of combination therapy, appropriate sequencing of treatments, and pharmacoeconomic outcomes of different therapies.

Immunotherapy recently entered the treatment paradigm of advanced HCC. Nivolumab is a fully human monoclonal antibody against programmed cell death protein‐1 (PD‐1) that disrupts checkpoint‐mediated inhibition of antitumor immunity. FDA approval has been obtained for use in the second‐line setting. In the phase I/II CheckMate‐040 study, nivolumab monotherapy demonstrated an ORR of 20% and a disease control rate of 64% with evidence of durable responses.^18^ In our cohort, four patients were treated with nivolumab after progression on bevacizumab; outcomes were variable. Investigation of combination therapy with anti‐VEGF treatment is ongoing.

Our findings suggest that bevacizumab is potentially a viable therapeutic option for advanced HCC. Further prospective study is needed to determine the efficacy of bevacizumab in advanced HCC and how to best incorporate anti‐VEGF therapy into treatment paradigms. Ongoing study of combination bevacizumab and checkpoint inhibition is of particular interest (NCT03382886, NCT02715531).

## CONFLICT OF INTEREST

Nothing to disclose.
